# Accessible and reliable neurometric testing in humans using a smartphone platform

**DOI:** 10.1038/s41598-023-49568-2

**Published:** 2023-12-18

**Authors:** H. J. Boele, C. Jung, S. Sherry, L. E. M. Roggeveen, S. Dijkhuizen, J. Öhman, E. Abraham, A. Uvarov, C. P. Boele, K. Gultig, A. Rasmussen, M. F. Vinueza-Veloz, J. F. Medina, S. K. E. Koekkoek, C. I. De Zeeuw, S. S. -H. Wang

**Affiliations:** 1 Princeton Neuroscience Institute, Princeton, USA; 2 Department of Neuroscience, Erasmus MC, Rotterdam, The Netherlands; 3 Department of Neuroscience, Vrije Universiteit, Amsterdam, The Netherlands; 4 Department of Clinical Sciences, Lund University, Lund, Sweden; 5 BlinkLab Pty, Sydney, Australia; 6 Department of Community Medicine and Global Health, University of Oslo, Oslo, Norway; 7 Department of Neuroscience, Baylor College of Medicine, Houston, USA; 8 Netherlands Institute for Neuroscience, Royal Academy of Arts and Sciences, Amsterdam, The Netherlands

**Keywords:** Classical conditioning, Neuroscience, Reflexes

## Abstract

Tests of human brain circuit function typically require fixed equipment in lab environments. We have developed a smartphone-based platform for neurometric testing. This platform, which uses AI models like computer vision, is optimized for at-home use and produces reproducible, robust results on a battery of tests, including eyeblink conditioning, prepulse inhibition of acoustic startle response, and startle habituation. This approach provides a scalable, universal resource for quantitative assays of central nervous system function.

## Introduction

Neurobehavioral assays of brain function can reveal fundamental mechanisms underlying neuropsychiatric conditions^1,2^, but typically require centrally located equipment in a laboratory test facility. Consequently, these tests are often unpleasant for participants as they require instruments attached to the face. Furthermore, fixed-lab testing cannot be used at scale in daily clinical practice.

We have developed a smartphone-based software platform, termed BlinkLab, to perform neurobehavioral testing free from facial instruments or other fixed-location equipment. This AI platform is designed to be used at home or in similar environments, independently or with the assistance of a caregiver, while following instructions from the mobile-device application. The tests include, but are not limited to, eyeblink conditioning, a form of sensory-motor associative learning, prepulse inhibition of the acoustic startle response, which measures the ability to filter out irrelevant information through sensorimotor gating, and startle habituation, which measures the ability for the intrinsic damping of repetitive stimuli.

**Figure 1:**
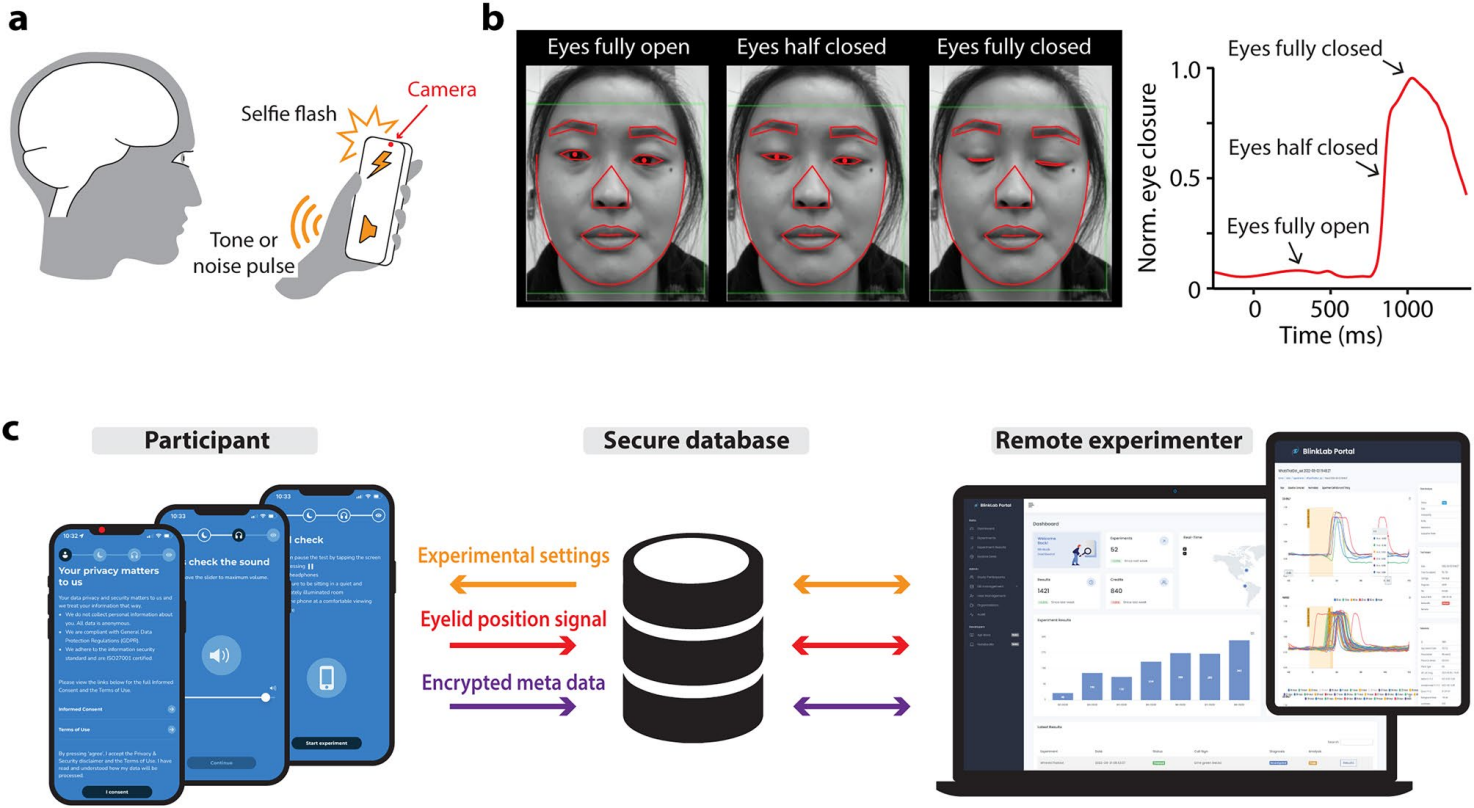
Architecture of smartphone-mediated neurobehavioral testing. (**a**) Auditory, visual, and tactile stimuli are delivered via the smartphone. The camera measures the participant’s responses at 60 Hz. (**b**) Facial landmark detection algorithms are capable of detecting eyelid movements in real-time on the smartphone. The participant gave informed consent for publication of these images in an online open-access publication. (**C**) The architecture of smartphone-mediated neurobehavioral testing includes a smartphone application (left), a secure database (middle), and a cloud-based analysis portal (right) that allows the remote experimenter to control experimental parameters and analyze collected data.

The BlinkLab application combines the smartphone’s ability to deliver stimuli and acquire data using computer vision with a secure cloud-based portal for data storage and analysis (Fig. 1). In our experiments, each audio and/or visual stimulus is presented with millisecond-precise control over parameters such as amplitude and frequency. In order to maintain participant attention, an entertaining movie of choice is shown with normalized audio levels. Participants’ responses are measured by the smartphone's camera and microphone, and are processed in real time using state-of-the art computer vision techniques, fully anonymized and transferred securely to the analysis portal.

## Materials and methods

### Subjects/participants

Participants were recruited from Princeton University (United States) and Erasmus MC (The Netherlands). For Princeton University, participants were invited using a SONA system (Sona Systems, Bethesda, USA). For Erasmus MC, participants were recruited using flyers and direct invites. There were no restrictions placed on sex at birth, self-identified gender, or race. Excluded were participants younger than 12 and those formally diagnosed with a neurodevelopmental, neuropsychiatric, or neurodegenerative condition. All participants gave informed consent to the study. Their participation was completely voluntary and they had the right to withdraw from the study at any time. The participants who are pictured in Fig. 1B, Supplementary Fig. 1, and the Supplementary Videos gave informed consent to publish these images in an online open-access publication. This study was approved by both the Institutional Review Board (IRB) for Human Subjects of Princeton University (IRB#13943) and the Medical Ethics Review Committee of Erasmus MC (# MEC- 2022-0116). All methods were performed in accordance with the relevant guidelines and regulations.

### Experimental setup

The smartphone application was developed in Swift, a compiled programming language developed by Apple. We chose to develop BlinkLab exclusively for iOS due to the uniformity and reliability of Apple's hardware and iPhone models, which simplified the app's development and ensured precise data collection.

The cloud-based analysis portal was developed in Symfony, a high-performance PHP framework for web development which uses PostgreSQL 14 as the database. Through an iterative development process, we enhanced the usability and impact of our app by continuously gathering data, validating functionality with a diverse user group, and refining algorithms for stimulus delivery and response (eyeblink) measurements. Our primary focus was on ensuring precision and maximum control in stimulus delivery by experimenters, emphasizing non-invasiveness. Moreover, we ensured that the system design adhered to established experimental protocols and scientific standards, underscoring our commitment to reliability and reproducibility. We also dedicated significant effort to making the testing experience pleasant for participants of all ages, aiming to maximize user engagement, by designing an intuitive and user-friendly app interface to optimize the setup of experiments, provide clear instructions, and facilitate data recording.

At the back-end, we implemented the option for researchers to fully design their own protocols for eyeblink conditioning, prepulse inhibition, and startle habituation on the platform. In fact, our platform allows researchers to conduct any neurometric test that involves smartphone stimulus delivery (auditory, visual, tactile) and measurement of facial responses, including eyeblinks. Combined with full featured multilingual questionnaire options before and after the tests, this opens up many more experimental applications as we standardly provide. Moreover, we implemented a secure system (TLS 1.3) to store experimental data while preserving features for data analysis and visualization within the app or for export of data that can be analyzed beyond the app.

The smartphone application was distributed using TestFlight and the AppStore. Eyelid movements were recorded with the smartphone’s front-facing camera at 60 frames per second. All stimuli were controlled by the BlinkLab app. The models of iPhones used were iPhone X, iPhone 11, iPhone 13 Pro, and iPhone 13 Pro Max. The headphones used were Pioneer over-ear wired headphones. The total length of a session was approximately 15 min for a single eyeblink conditioning session, 12 min for a prepulse inhibition session, and 5 min for a startle habituation session (actual length depended on the total durations of the random intertrial intervals).

Prior to the start of the test, we assured that the participant was in a quiet environment with ambient lighting and in a comfortable position. The app continuously monitored the surrounding environment, including the light intensity using the camera, the background noise using the microphone, and the smartphone position and movements using the accelerometer. If one of the aforementioned values was out of bounds, the video paused and the app instructed the user on how to change the environment or to try the test at another time. During the experiment, the user watched an entertaining (audio normalized) movie while the stimuli for eyeblink conditioning, prepulse inhibition, or startle habituation were delivered. For each trial, facial landmark detection algorithms were used to track and record the position of the participant’s facial landmarks over time to determine amplitude and timing of the eyelid closure. Users could see a small progress bar at the bottom of the screen that showed them how far along they were in the experiment. All results were securely transferred and stored in a cloud-based analysis environment where data became immediately accessible for researchers through the BlinkLab analysis portal.

To address the potential influence of strong emotional content on test outcomes, participants in the current manuscript were restricted to selecting movies solely from the sitcom category (e.g., “The Office” and “Friends”) or nature documentaries. For the current study, participants watched the following movies: Eyeblink Conditioning: Coco, n = 30 views; Friends—Season 1, n = 53 views; The Office—Seasons 1, 2, 3, n = 85 views; Nature documentary—Deserts to Grasslands, n = 2 views. Prepulse inhibition of Startle: Coco, n = 4 views; Nature documentary—Deserts to Grasslands and The Lost Forest, n = 2 views; Friends—Season 1, n = 7 views; Modern Family—Season 9, n = 1 view; Oddbods, n = 1 view; The Office—Seasons 3 and 7, n = 12 views; Pokemon, n = 1 view; Super Mario Bros, n = 1 view; Toy Story 4, n = 1 view. Startle habituation: Masha et Michka, n = 1 view; The Office—Seasons 3 and 7, n = 13 views; Paw Patrol, n = 2 views. While we acknowledge that we cannot entirely rule out the potential influence of video content at an individual level, it is crucial to note that the primary objective of the current manuscript is to demonstrate the concept for smartphone-based eyeblink conditioning and other psychometric testing, rather than a full examination of variables that can influence the test outcomes.

In general, both raw and processed data are available in the most widely used data standards, as well as through BlinkLab’s cloud-based analysis and visualization tools. The BlinkLab platform includes a comprehensive ‘export/download data’ feature within the online portal. This functionality allows researchers to access and export raw data of the 77 extracted facial landmarks that are extracted from the raw videos (frame rate at 60 Hz). Users can selectively filter and aggregate data based on numerous variables, including subject demographics, diagnoses, age, sex, iPhone model, and any other custom tag defined by the researcher. The data can be aggregated for any specific research demand and exported in a range of formats, like CSV and other data standards, to ensure compatibility with a broad spectrum of data analysis tools, such as R, MatLAB, Python, and SPSS.

### Eyeblink conditioning training paradigm

Participants (n = 14, details in Table S2) completed 6–9 eyeblink conditioning sessions within a 14-day span, with no more than 2 sessions per day. The conditioned stimulus (CS) consisted of a white circular dot, 1 cm in diameter, in the middle of the screen which lasted for 450 ms. The unconditioned stimulus (US) consisted of a simultaneous 105 dB, 50 ms white noise pulse and a 50 ms full screen retina flash. The CS and US were presented in a delay paradigm, which means that the CS and US have a delay in onset, but temporally overlap and co-terminate at the end. For session 1–6, the interval between CS and US onset was set at 400 ms (Supplementary Fig. 2). The interval between the trials was set randomly between 7 and 20 s. During sessions 1–6, participants received a total of 50 trials distributed over 5 blocks. Each block consisted of 1 CS-only, 1 US-only, and 8 paired CS-US trials, semi-randomly distributed throughout the block. In session 7, participants received a short CS (100 ms) in CS-only trials to demonstrate cerebellum-dependent response timing. In sessions 8 and 9, the duration of the interstimulus interval (ISI) was suddenly extended to 700 ms. The longer ISI of 700 ms was used to assess the level of response timing adaptability. A training session lasted for about 15–20 min.

### Prepulse inhibition training paradigm

Participants (n = 30, details in Table S2) completed a single prepulse inhibition session. One session contained 55 trials. The two stimuli were defined as: 1) a pulse consisting of a 105 dB, 50 ms white noise audio burst and 2) a prepulse, consisting of a 50 ms white noise audio burst of varying amplitude that was always softer than the pulse. We used prepulses at four intensities: 65 dB, 75 dB, 83 dB and 93 dB. First, a total of 5 habituation trials containing white noise bursts of various soft intensities were presented. This allowed for the participant to relax and settle into the movie. After these 5 trials, 10 blocks of 5 trials were presented. Each block consisted of a pulse-only trial and 4 prepulse-pulse trials with prepulse amplitudes of consecutively 5, 10, 25 and 50% of the startle amplitude. A prepulse always preceded the pulse by 120 ms (Supplementary Fig. 3). Intertrial interval (ITI) was set at random between 10 and 25 s. A training session lasted for about 15–20 min.

### Startle habituation training paradigm

Participants (n = 14, details in Table S2) completed a single startle habituation session. One session contained 10 trials. For startle habituation, we used a 1.33 Hz pulse train of five white noise audio bursts at an intensity of 105 dB (Supplementary Fig. 4). The ITI was set at random between 20 and 40 s. A training session lasted for about 15–20 min.

### Data analysis

We extracted the facial bounding box (Fig. 1b), using the XYZ coordinates, which denotes the three-axis readings from the smartphone's gyroscope to measure the device's orientation along the x, y, and z axes. These orientation data are crucial, as it informs our software of the smartphone's position relative to the eye when calculating the eyelid position signals, ensuring that the coordinates of the landmarks around the eye are accurately captured regardless of how the phone is held. Eyelid position signals were calculated in real-time on the smartphone based on the x–y coordinates, relative to the facial bounding box, of the six landmarks around the eye. For this, we calculated the difference between the y position values of the sum of the two upper eyelid landmarks and the sum of the two lower eyelid landmarks with the x position values of the two eye corner landmarks (Supplementary Fig. 1a). For each type of trial, a single snippet was taken from the video of the eyelid position signal hereafter called ‘eyeblink trace’. Individual eyeblink traces were analyzed with custom computer software (R Studio; Boston, MA, v1.3.1093). Eyeblink traces were filtered in forward and reverse directions with a low-pass Butterworth filter using a cut-off frequency at 50 Hz. Eyelid traces were then normalized for each session on a scale from 1 (representing a full blink) to 0 (indicating the eye is fully open). This normalization approach aligns with standard practices in mouse eyeblink conditioning using a camera system^3–7^. Yet, unlike conventional mouse eyeblink systems employing a puff of air to induce a full eyelid closure, where the amplitude of US-evoked blinks is typically considered, we opted for a slightly different approach. Since blinks elicited by the brief white noise pulse in our smartphone app were occasionally partial rather than full closures, we included spontaneous, full eyeblinks in our analysis. These spontaneous blinks were captured during the 500 ms pre-stimulus baseline periods. The amplitude of these spontaneous blinks was set at 1 and the pre-stimulus baseline periods were standardized, with their amplitude aligned at 0 (Supplementary Fig. 1b).

### Eyeblink conditioning

To quantify eyeblink conditioning, we used four outcome measures: (1) The conditioned response (CR) amplitude, defined as the amplitude of the eyelid response in the 60 ms—US offset window; (2) the CR percentage, defined as the percentage of trials within a session that contained a CR, whereby a CR was defined as an eyelid movement larger than 0.15 and with a latency to CR peak between 60 ms after CS onset and US offset; (3) the latency to CR onset in ms after CS onset, and (4) the latency to CR peak in ms after CS onset. The calculation of the CR percentage, CR amplitude, and latency to CR onset included both paired CS-US trials and CS-only trials. Including all 55 trials, rather than just the five CS-only trials, allowed for a more accurate estimate of the actual learning at a subject level. It is essential to note that our focus was solely on the interval before the onset of the US, and the eyeblink responses in that interval are inherently anticipatory (i.e., conditioned) and cannot be elicited by the US. For latency to CR peak, we only included CS-only trials since they show the full kinetic profile of the eyeblink CR and provide a better estimate of the adaptive timing of eyeblink CRs. Previous human studies employing a camera system for eyeblink conditioning used a threshold of ~50% eye closure^8^ or did not specify the threshold for defining a blink^9^. Consistent with previous camera-based mouse studies^3–7^, we found that a fixed threshold of 0.15 was sufficient to minimize false-positive CRs.

### Prepulse inhibition

The response detection window for latency to the peak of eyelid responses to the pulse was set at 60–330 ms after pulse onset^10,11^. After careful data inspection, we found that the typical alpha startle response in humans using our smartphone system had a latency to peak between 60 and 330 ms after pulse onset. For that reason, we used these intervals to capture both the R1 and R2 components of the startle blink (Supplementary Fig. 3, 4). This latency was corrected for potential inherent latencies because of the use of Bluetooth. Please note that this latency is slightly longer than the latencies traditionally used in EMG studies. The reason for this is that EMG directly measures muscular activity and our video approach measures the resulting movement (muscle activity precedes movement)^12^. To analyze amplitude reduction, we used the amplitude of the normalized eyelid closure at the mean peak time of significant startle responses calculated over *all* trials. In addition to responsiveness to the startle stimuli, we analyzed the effect of the prepulse itself on normalized eyelid closure in a similar fashion, but here we used a response window of 60–180 ms after the prepulse.

### Startle habituation

The response detection windows for startle responses were set at 60–330 ms after a startle stimulus^10,11^. Response detection was done in a similar fashion as described above for eyeblink conditioning and prepulse inhibition (Supplementary Fig. 4). To analyze amplitude reduction, we used the amplitude of the normalized eyelid closure at the mean peak time of significant startle responses calculated over *all* trials.

### Statistical analysis

Statistical analysis and data visualizations were done in R Studio (V2022.02.03) using the following packages: dplyr, emmeans, ggplot2, lmerTest, nlme, tidyr, and tidyverse. We used multilevel linear mixed-effects (LME) models in R Studio because they are more robust to violations of normality assumptions, which is often the case in biological data samples. LME models can better accommodate the nested structure of our data (i.e., trial nested within session, session nested within subject, subject nested within group) and prevent data loss by using summary measures. As an added benefit, LME models are better at handling missing data points than repeated measures analysis of variance (ANOVA) models and do not require homoscedasticity as an inherent assumption. In our LME, we used session as a fixed effect and subject as a random effect. Goodness-of-fit model comparison was determined by evaluating log likelihood ratio, BIC, and AIC values. The distribution of residuals was inspected visually by plotting the quantiles of standard normal versus standardized residuals (i.e. Q–Q plots). Data were considered as statistically significant if the p-value was less than 0.05.

## Results

Software development was done in Swift, a compiled programming language developed by Apple. This initial effort used iOS due to the uniformity and reliability of Apple's hardware and iPhone models, which simplified software development and ensured precise data collection. For optimal use, prior to the start of each test, we assured that the participant was in a quiet environment with ambient lighting and in a comfortable position. The app continuously monitored the surrounding environment, using the camera to monitor light intensity, the microphone to detect background noise, and the accelerometer to measure smartphone position and movement. If any of the aforementioned values went out of bounds, the app paused the video and instructed the user how to continue or try the test at another time. The testing experience was made pleasant by providing clear instructions, using an intuitive and user-friendly interface, and during the testing itself, showing the participant an entertaining (audio level normalized) movie while stimuli for eyeblink conditioning, prepulse inhibition or startle habituation are delivered. For each trial, facial landmark detection algorithms were used to track and record the position of the participant’s facial landmarks over time to determine amplitude and timing of the eyelid closure. All results were securely transferred and stored in a cloud-based analysis environment where data became immediately accessible for researchers through the BlinkLab analysis portal.

**Figure 2:**
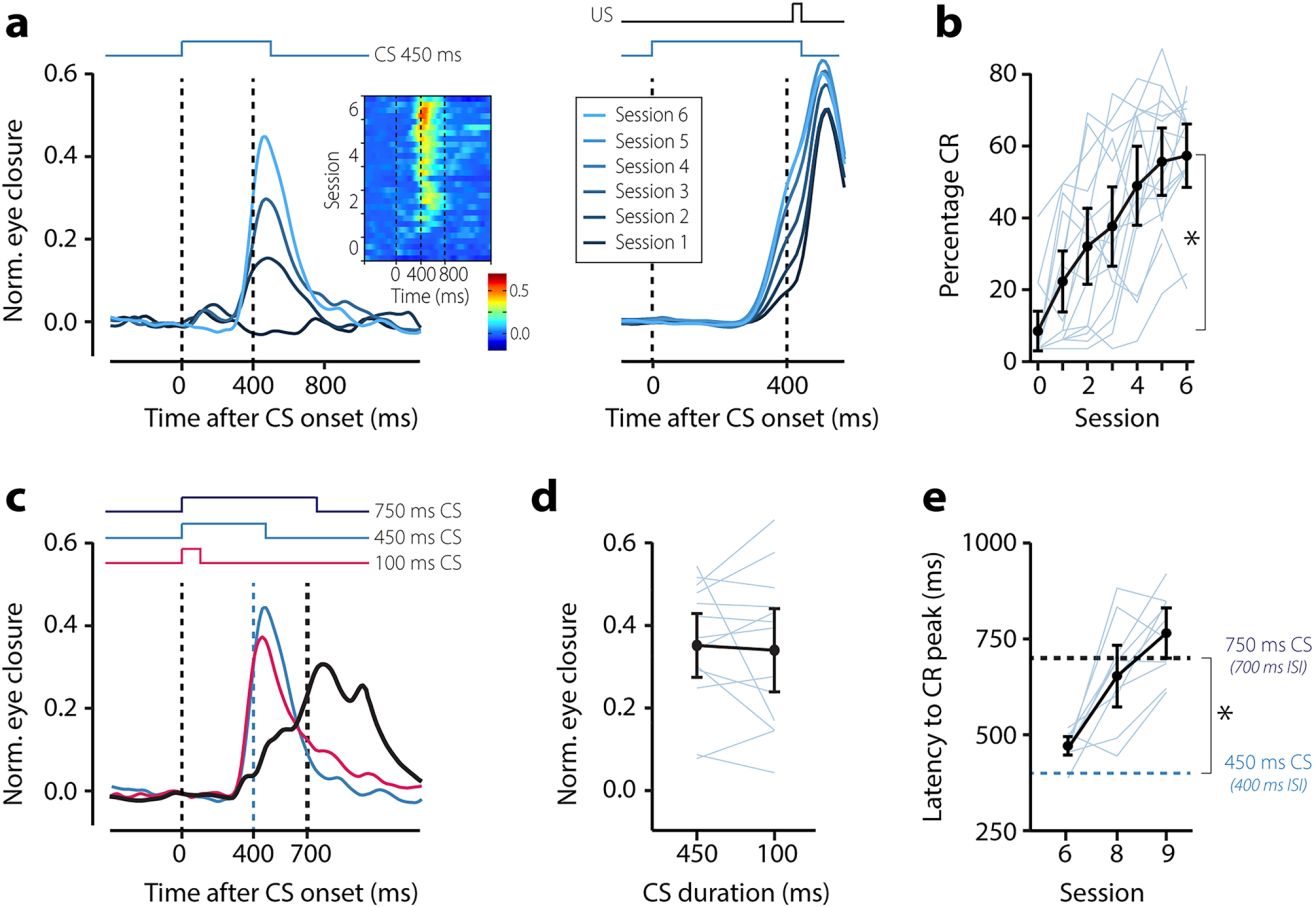
Smartphone-mediated delay eyeblink conditioning. (**a**) Session averaged eyeblink traces of CS-only trials (left) and paired CS-US trials (right). Note the gradual acquisition of eyeblink conditioned responses. The first vertical dashed line indicates the onset of the CS, the second one indicates the onset of the US. The heatmap shows the average per trial for CS-only trials during the 6 acquisition sessions; heat represents the amplitude of eyelid response. (**b**) Percentage of CRs per session. Light blue lines are individual learning curves, the thick black line represents the group average. (**c**) Session averaged eyelid CRs in response to a CS with a duration of 450 ms (light blue trace) at the end of acquisition (session 6), a short probe CS with a duration of 100 ms (red trace), and a long CS with a duration of 750 ms (dark blue trace). The short CS was never reinforced with the US. The long CS was reinforced with a US. (**d**) The short CS of 100 ms elicited eyeblink CRs that were indistinguishable from those evoked by the original 450 ms CS. (**e**) As a result of the ISI switch, the latency to CR peak shifted from the onset of the old US at 400 ms to the onset of the new US at 700 ms. 100 ms CS, Conditional stimulus with a duration of 100 ms; 450 ms CS, Conditional stimulus with a duration of 450 ms (ISI 400 ms); 750 ms CS, Conditional stimulus with a duration of 750 ms (ISI 700 ms); CR, Conditioned response. All error bars represent 95% confidence intervals.

Eyeblink conditioning using the smartphone approach induced conditioned responses comparable with traditional stimuli such as an airpuff^13^. The unconditioned stimulus (US) was a 50 ms white noise pulse paired with a brief screen flash, which reliably elicited a reflexive eyelid closure and activated cerebellum-dependent learning mechanisms^14,15^. The conditioned stimulus (CS) was a white 1 cm circular dot presented for 450 ms as an overlay over the movie at the screen’s center (See online materials). Repeated pairings of the CS and US in a delay paradigm on the smartphone, with an interstimulus interval (ISI) of 400 ms, resulted in a robust acquisition of eyelid conditioned responses (CR) at the end of six sessions (acquisition phase) of fifty paired CS-US trials each. The CR amplitude increased from -0.02 (± 0.05 95% CI) in baseline session 0 to 0.35 (± 0.09 95% CI) in session 6 (F (6,4044) = 74.82, *p* < 0.001, LME) (Fig. 2a, Table S2). Similarly, the CR percentage increased from 5.3 (± 6.6 95% CI) in baseline session 0 to 58.5 (± 10.6 95% CI) in session 6 (F (6,4044) = 65.13, *p* < 0.001, LME)) (Fig. 2b, Table S2). CR timing significantly improved over the course of training, with session 6 yielding CRs with a latency to peak around the onset of the expected US at 470.46 (± 26.53 95% CI) ms after CS onset (F (6,270) = 8.92, *p* < 0.001, ANOVA on LME). To confirm the putative cerebellar nature of these conditioned responses, we performed two additional tests after the acquisition phase. First, we tested the effect of a probe CS that was relatively short (i.e., only 100 ms) compared to the one that was used during training (session 7). This probe CS was only presented in CS-only trials and never reinforced with a US. In line with previous reports^16,17^, we found that this short probe CS was able to elicit normal CRs in terms of CR percentage (F(1,1169) = 0.57, *p* = 0.450) and CR amplitude (F (1,1169) = 0.68, *p* = 0.41, LME) (Fig. 2c,d, Table S2). Second, we tested the effect of an extension of the interstimulus interval from 400 to 700 ms during two additional training sessions (sessions 8 and 9). We found that the timing of eyeblink CRs adapted to the new longer ISI with a new latency to CR peak of 765.6 (± 75.85 95% CI ms) (Fig. 2c,e, Table S2) (F (2,113) = 42.21, *p* < 0.001, ANOVA on LME), consistent with previous studies on eyeblink conditioning^18^. As a result of our smartphone approach, we were able to induce robust learning in the eyeblink conditioning paradigm and produce data that had less variability in learning curves previously reported, including in our own studies on human eyeblink conditioning (compare our smartphone approach with for instance^13,20^). The overall learning pattern now closely resembles those described previously in mouse and rabbit eyeblink conditioning literature^3,21^, with a smooth, gradual increase of the CR amplitude, percentage, and timing that is adaptive to the interstimulus interval.

**Figure 3:**
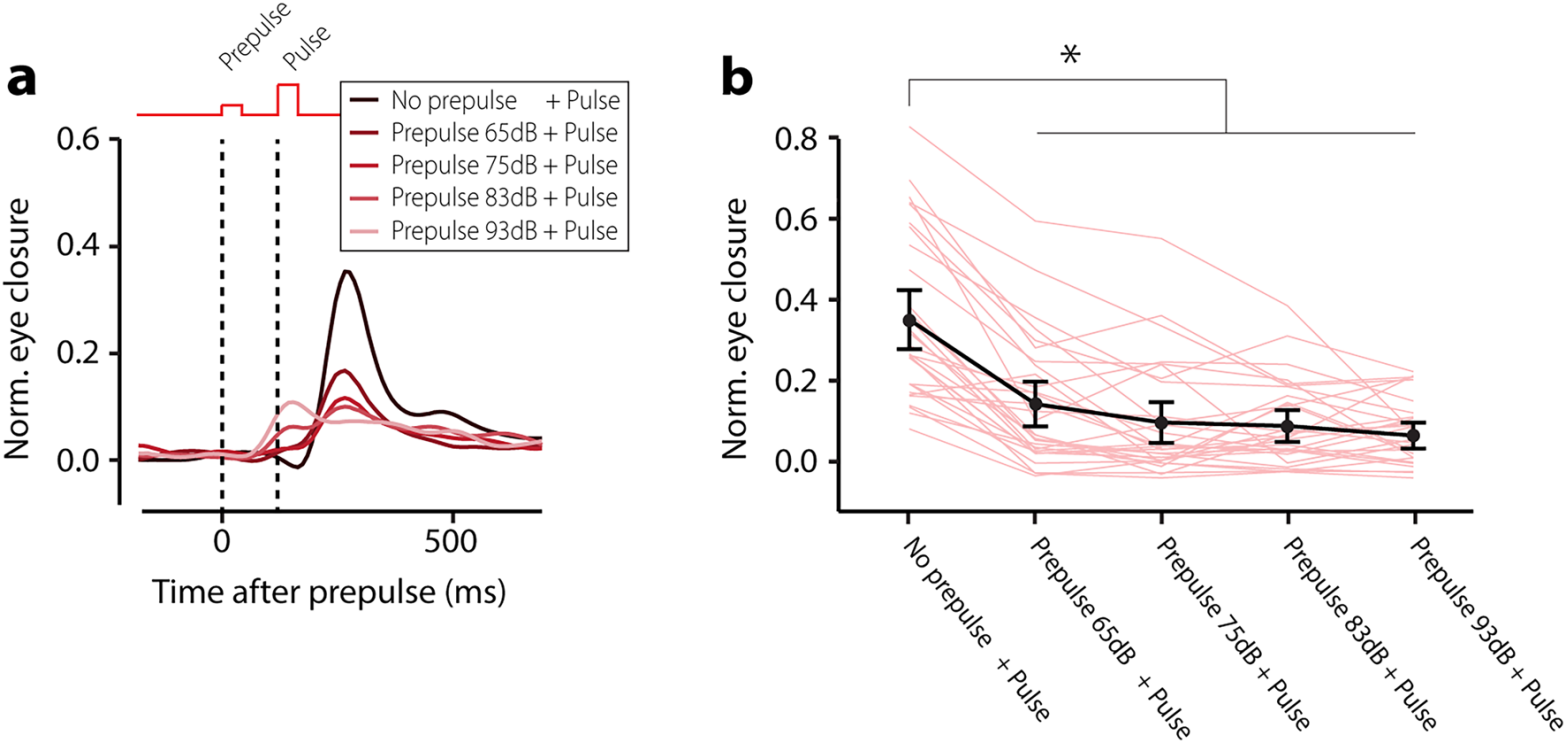
Smartphone-mediated prepulse inhibition of the acoustic startle response. (**a**) Averaged eyelid traces for the different trial types. The presentation of a soft sound (prepulse, first vertical dashed line) 120 ms before the loud sound (pulse, second vertical dashed line) resulted in a significant inhibition of the acoustic eyelid startle response. Note that higher prepulse intensities (25% and 50%) start to evoke a small startle response to the prepulse. (**b**) Amplitude of eyelid closure as a function of trial type. All error bars represent 95% confidence intervals.

Next, we studied prepulse inhibition of the startle response, using a 50 ms white noise audio burst at 105 dB as the pulse and a similar burst ranging between 75 and 95 dB as the prepulse. The ISI was set at 120 ms (See online materials). Eyelid startle responses in the pulse-only trials had an average amplitude of 0.35 (± 0.08 95% CI), while responses in trials where the pulse was preceded by the weaker prepulse ranged between 0.07 (± 0.03 95% CI) and 0.14 (± 0.06 95% CI) (Fig. 3 Table S3). We found a significant main effect of trial type (F (4,1434) = 99.44, *p* < 0.001, ANOVA on LME) and pairwise post-hoc testing revealed that the largest effects were present between the pulse and the prepulse + pulse trials (Fig. 3, Table S3). With prepulse inhibition values of about 70% (100-(0.10/0.35) * 100), our data show a strong inhibition of the acoustic startle responses (compare our results for instance with^22–24^).

**Figure 4:**
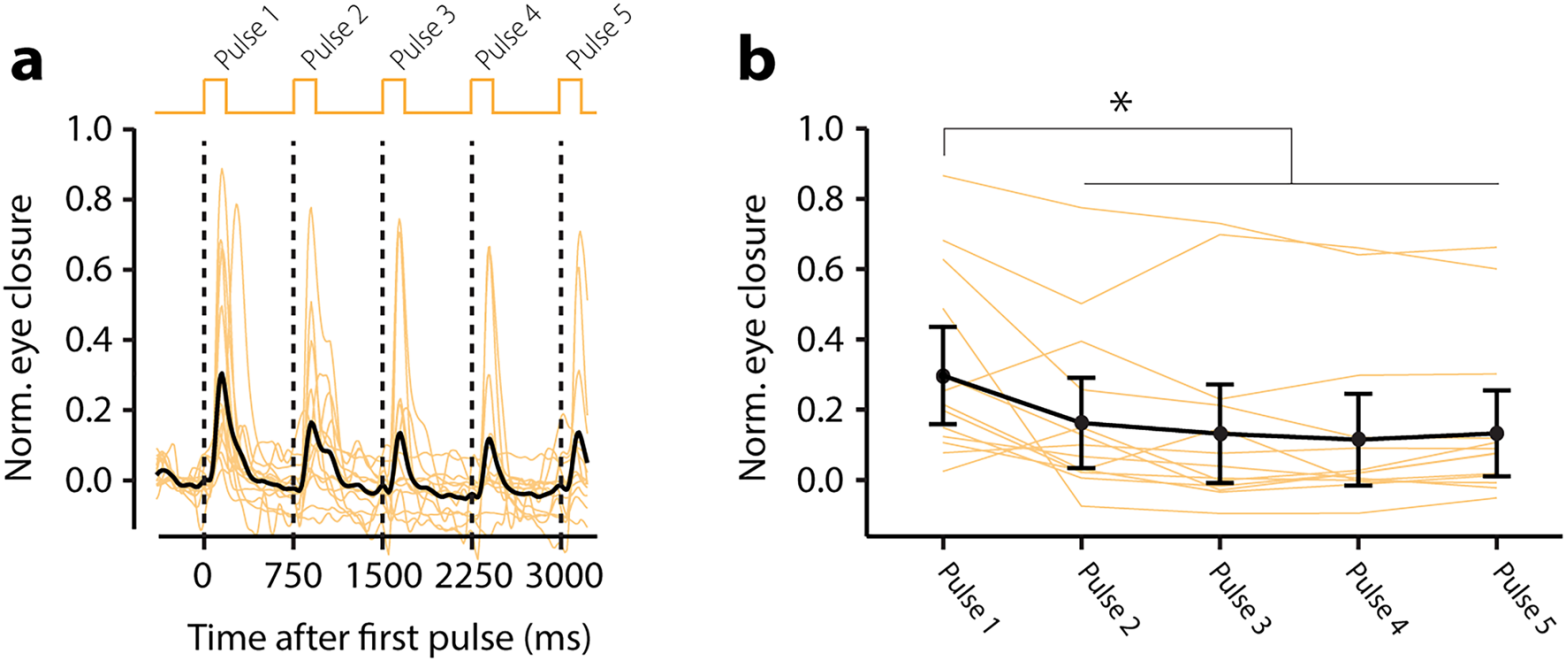
Smartphone-mediated startle habituation. (**a**) Individual (yellow) and group averaged (black) eyelid traces during the presentation of 5 consecutive acoustic white noise pulses. (**b**) Amplitude of eyelid closure as a function of trial type. Notably, the most pronounced habituation occurs between pulse 1 and pulse 2. Subsequent to pulse 2, there was minimal habituation observed in neurotypical people when employing a fixed 750 ms interstimulus interval. Error bars represent 95% confidence intervals.

Finally, we studied startle habituation using a 1.33 Hz pulse train of five white noise audio bursts at an intensity of 105 dB. We found a significant reduction of the eyelid startle amplitude over the course of the stimulus pattern, starting with 0.30 (± 0.15) at the first pulse and 0.13 (± 0.14) at the fifth pulse (Fig. 4, Table S4, F (4,612) = 18.14, *p* < 0.001, LME). Post-hoc testing revealed significant effects between pulse 1 and any of the other pulses (Table S4). The most pronounced habituation occurred between pulse 1 and pulse 2. Subsequent to pulse 2, there was no significant further reduction in eyelid startle amplitude.

## Discussion

Together, our data show a proof-of-principle that we can perform well-established neurobehavioral testing using accessible smartphone technology. In contrast to conducting these tests in a laboratory environment, we found that people performed better and had less variability in their performance by doing them on a smartphone in a comfortable home-like environment. Since these tests are reflex-based and do not require verbal or social interaction, they allow for large-scale cross-cultural human studies and cross-species translational research.

While a diverse array of camera systems is available for the measurement of eye movements and blinks, the utilization of such technologies in tracking eyelid movements during eyeblink conditioning and startle modulation experiments is limited in the current literature^8,9^. Today, electromyography (EMG) remains the preeminent modality for investigating eyeblink conditioning and startle modulation in human subjects^25–27^. This preference for EMG is attributable, in part, to its historical efficacy in detecting subtle eyelid movements compared to computer vision algorithms. Furthermore, the customary delivery of a blink-inducing puff of air to the eye necessitates facial tubing, and the concurrent installation of EMG wires incurs minimal additional effort. Our data substantiate the viability of using the smartphone’s camera and employing a non-somatosensory stimulus, distinct from an air puff, to elicit a reflexive eyelid closure and engage cerebellum-dependent learning mechanisms^14,15^.

For robust and reproducible data acquisition, our smartphone-based platform enables researchers to measure variables that could potentially influence the test outcomes, such as time of the day that the data was collected. In addition, the smartphone platform allows researchers to design pre- and/or post-test questionnaires using various styles (e.g., dropdown, checkmarks, or open questions). Typical pre-test questions would include the participant's sleep status, use of medication, level of physical activity, and degree of stress, among others. Examples of post-test questions include the user's experience with the app, the subjective loudness of stimuli, and the perceived intensity of flashes. It should be noted that these variables were not taken into account for the current proof-of-concept study.

Performance in eyeblink conditioning, prepulse inhibition, and startle habituation is strongly correlated with neuropsychiatric conditions, including autism^1,13^, schizophrenia^28,29^, dementia^13,31^, Parkinson’s^32^, and Huntington’s disease^32–34^. As such, these tests have repeatedly been suggested as a potential biomarker to diagnose and monitor (pharmaceutical) intervention of neurodevelopmental and neurodegenerative conditions. Since the new smartphone approach does not require unpleasant in-lab testing while providing consistent data with reduced variability, it opens up the possibility of using these quantitative tests in clinical practice.

### Supplementary Information

Supplementary Video 1.

Supplementary Video 2.

Supplementary Video 3.

Supplementary Video 4.

Supplementary Video 5.

Supplementary Information 1.

## Data Availability

Complete eyeblink conditioning dataset, prepulse inhibition dataset, and startle habituation dataset are publicly available at: https://github.com/506574657220426F656C650D/POC-datasets. Data is anonymized, the unique identifier for the different subjects is the subject_id.

## Code availability

Complete R syntax for data analysis is available at: https://github.com/506574657220426F656C650D/POC-datasets.
