# Supplementary material for: Accessible and reliable neurometric testing in humans using a smartphone platform
Source: Sci Rep. 2023 Dec 18;13:22871. doi: 10.1038/s41598-023-49568-2 (PMC10739701; doi:10.1038/s41598-023-49568-2)
Supplement: Supplementary file 6 — Supplementary Information 1. [file 41598_2023_49568_MOESM6_ESM.docx]

Supplementary materials for:

**Accessible and reliable neurometric testing in humans using a smartphone platform**

H.J. Boele^1,2,^*, C. Jung^1^, S. Sherry^1^, L.E.M. Roggeveen^2^, S. Dijkhuizen^2^, J. Öhman^3^, E. Abraham^1^, A. Uvarov^4^, C.P. Boele^2^, K. Gultig^2^, A. Rasmussen^3^, M.F. Vinueza-Veloz^2, 5^, J.F. Medina^6^, S.K.E. Koekkoek^2^, C.I. De Zeeuw^2, 7^, S.S.-H. Wang^1,^*

^1^ Princeton Neuroscience Institute, Princeton, USA

^2^ Department of Neuroscience, Erasmus MC, Rotterdam, The Netherlands

^3^ Department of Clinical Sciences, Lund University, Sweden

^4^ BlinkLab Pty LTD, Sydney, Australia

^5^ Department of Community Medicine and Global Health, University of Oslo, Oslo, Norway

^6^ Department of Neuroscience, Baylor College of Medicine, USA

^7^ Netherlands Institute for Neuroscience, Royal Academy of Arts and Sciences, Amsterdam

*Co-corresponding authors: [hboele@princeton.edu](mailto:hboele@princeton.edu), [sswang@princeton.edu](mailto:sswang@princeton.edu)

**
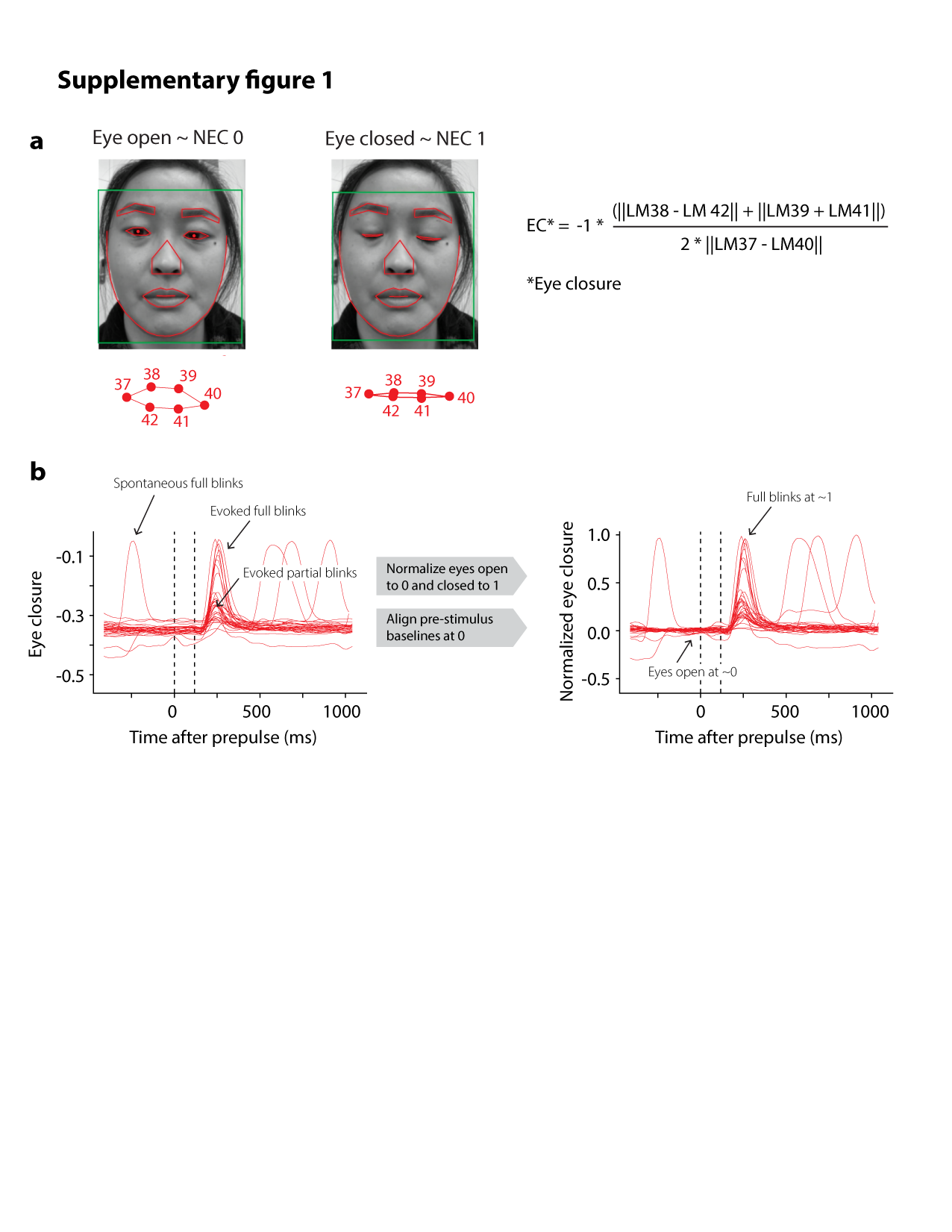
**

**Supplementary Figure 1 | Computer vision algorithms were used to capture facial landmarks detection, including those of the eyelids. (a)** Eyelid closure (EC) was calculated for each eye, using six landmarks for each eye. The participant gave informed consent for publication of these images in an online open-access publication. **(b)** Eyelid traces were normalized for each session on a scale from 1 (representing a full blink) to 0 (indicating the eye is fully open). Spontaneous blinks were captured during the 500 ms pre-stimulus baseline periods. The amplitude of these spontaneous blinks was set at 1 and the pre-stimulus baseline periods were standardized, with their amplitude aligned at zero.

**
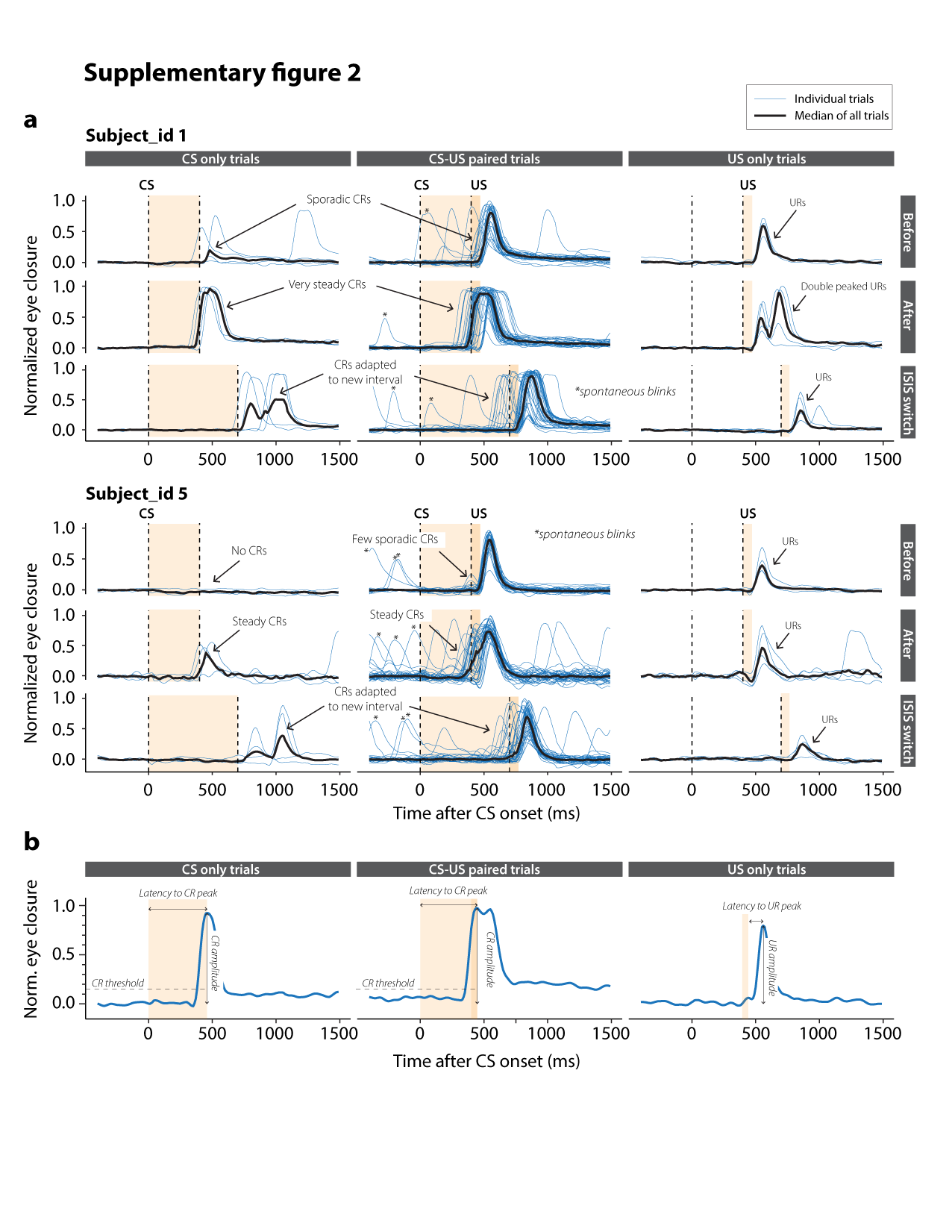
**

**Supplementary Figure 2 | Raw eyelid traces of two participants (Subject_id 1 and 5) for eyeblink conditioning.** Thin blue traces are individual trials, thick black trace is the session median. All data is included, no traces were deleted from these datasets. **(a)** Top panel: during session 1, the participants show no or only a few sporadic responses to the conditional stimulus (CS, white dot) and reflexive eyeblinks to the unconditional stimulus (US, noise burst). Middle panel: during session 6, both participants show steady responses to the CS, which are called conditioned responses (CRs). Bottom panel: extending the interval between the CS and US from 400 to 700 ms, results in adaptation of the eyeblink CRs. **(b)** Explanation of how we extracted the eyeblink conditioning outcome measures at a single trial level. For each trial (blue trace) we calculated the CR amplitude, the CR percentage based on 0.15 threshold, and the latency to CR peak.

**
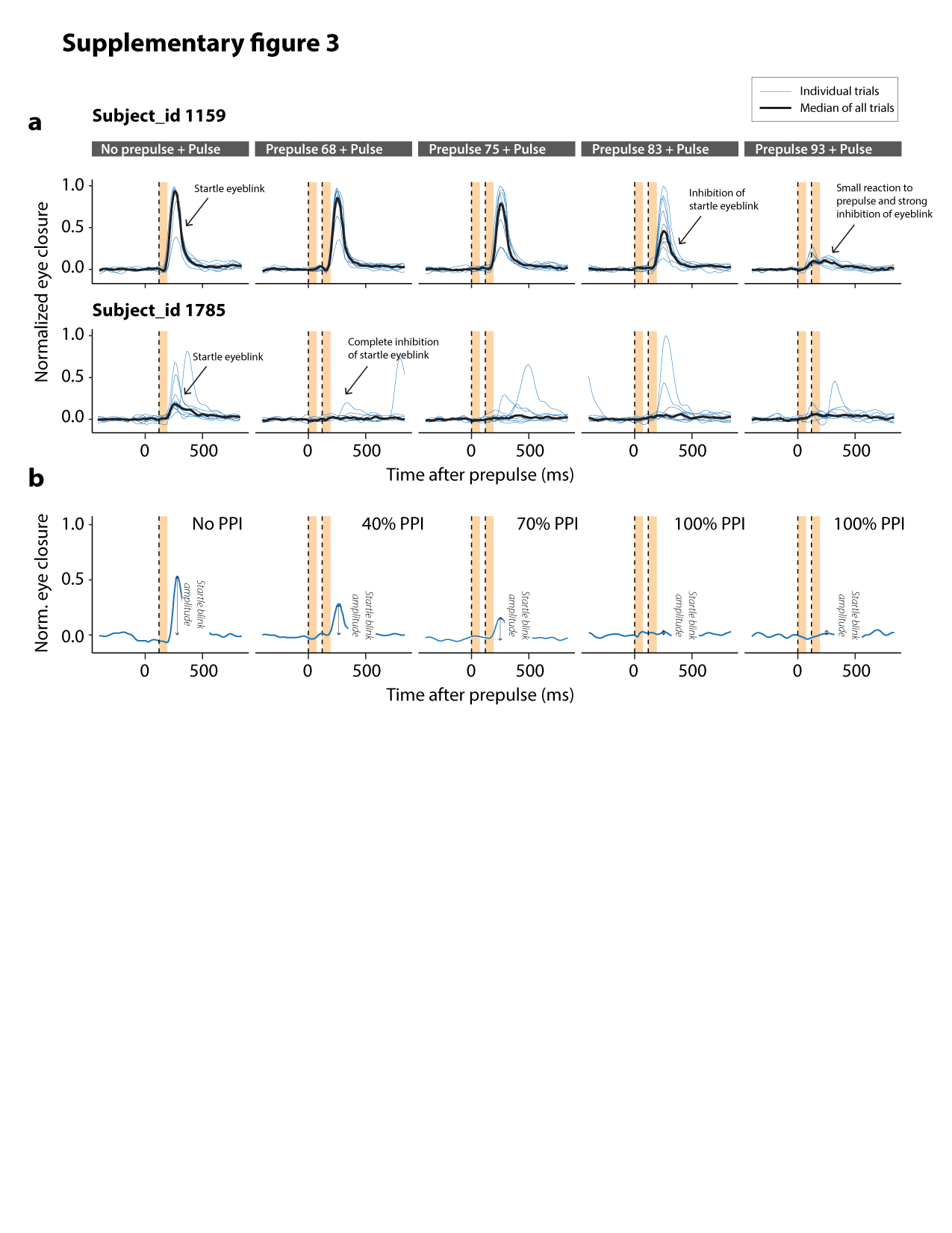
**

**Supplementary Figure 3 | Raw eyelid traces of two participants (Subject_id 1159 and 1785) for prepulse inhibition of the acoustic eyelid startle reflex.** Thin blue traces are individual trials, thick black trace is the session median. All data is included, no traces were deleted from these datasets. **(a)** Normalized eyelid responses in the five different trial types. Left panel shows the pulse only trials at 105 dB; the other panels show the prepulse + pulse trials, with the prepulse at different intensities. Note that a louder prepulse often leads to a stronger inhibition. **(b)** Explanation of how we extracted the prepulse inhibition outcome measures at a single trial level.


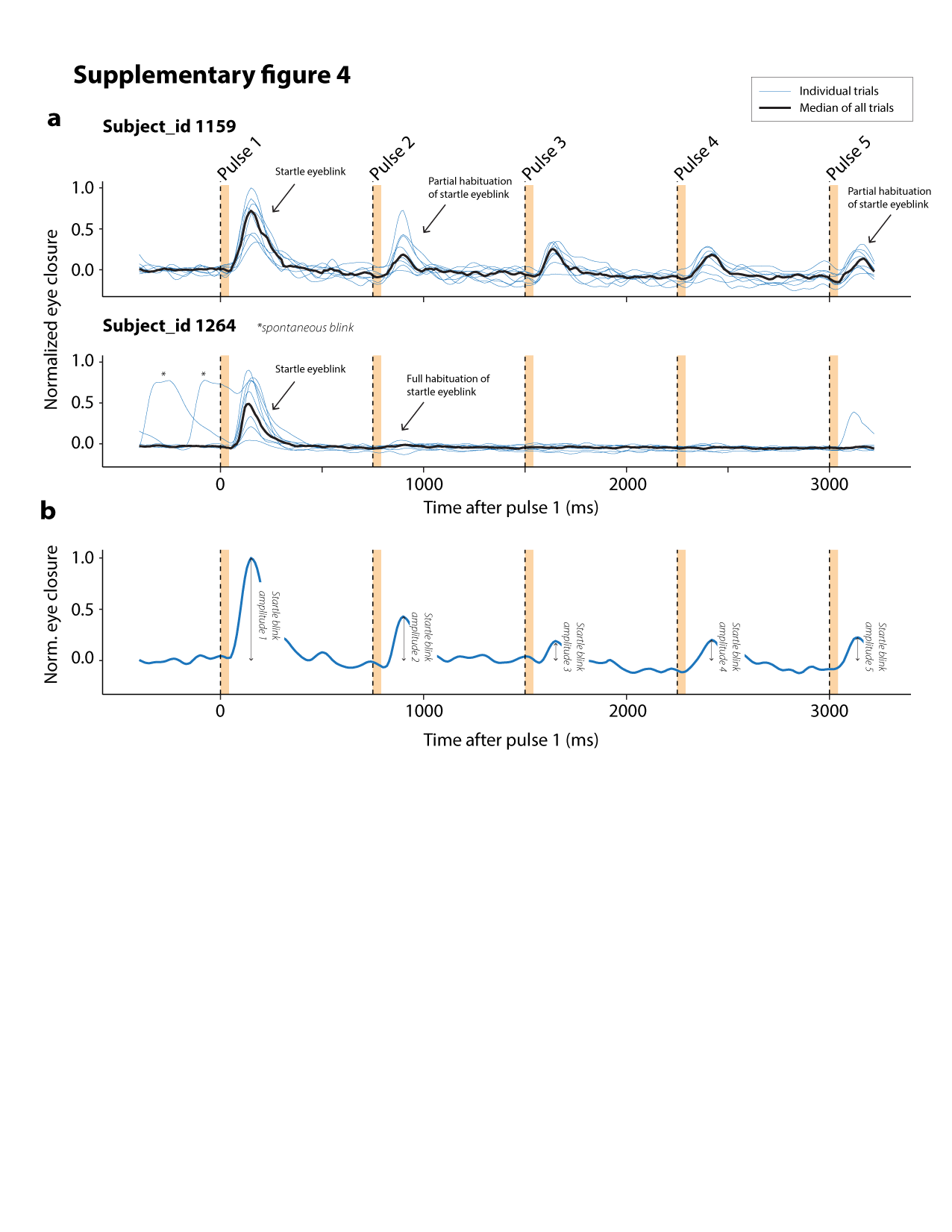


**Supplementary Figure 4 | Raw eyelid traces of two participants (Subject_id 1159 and 1264) for startle habituation.** Thin blue traces are individual trials, thick black trace is the session median. All data is included, no traces were deleted from these datasets. **(a)** Note that in both participants the strongest habituation takes place between pulse 1 and 2. **(b)** Explanation of how we extracted the startle habituation outcome measures at a single trial level.

| **Supplementary table 1 \| Participants per neurometric test** | | | |
| --- | --- | --- | --- |
|  |  |  |  |
|  | **Eyeblink conditioning (n = 14)** | **Prepulse inhibition (n = 30)** | **Startle habituation (n = 14)** |
| **Age (years)*** | 29 (± 13.17) | 25 (± 13.82) | 34 (± 17.87) |
| **Sex** |  |  |  |
| Male | 5 (36%) | 14 (47%) | 6 (43%) |
| Female | 9 (64%) | 16 (53%) | 8 (57%) |
|  |  |  |  |
| ** All values: mean ± 1 standard deviation* | | | |

| **Supplementary table 2 \| Pavlovian eyeblink conditioning (n = 14 participants)** | | | | | | | | |
| --- | --- | --- | --- | --- | --- | --- | --- | --- |
|  |  |  |  |  |  |  |  |  |
| **Session** | **Phase** | **ISI duration** | **CS duration** | | **CR percentage** | **CR amplitude** | **Latency to CR onset (ms)** | **Latency to CR peak (ms)** |
|  |  |  | ***CS-US*** | ***CS only*** | **(mean ± 95% CI)** | **(mean ± 95% CI)** | **(mean ± 95% CI)** | **(mean ± 95% CI)** |
| 0 | Baseline | 400 ms | 450 ms | 450 ms | 5.3 (± 6.6) | -0.02(± 0.05) | 156.33(± 121.24) | 229(± 115.18) |
| 1 | Acquisition | 400 ms | 450 ms | 450 ms | 20.4 (± 10.0) | 0.08(± 0.07) | 163.47(± 34.78) | 412.5(± 96.4) |
| 2 | Acquisition | 400 ms | 450 ms | 450 ms | 31.0 (± 12.5) | 0.15(± 0.08) | 179.11(± 27.3) | 531.96(± 31.71) |
| 3 | Acquisition | 400 ms | 450 ms | 450 ms | 37.0 (± 13.1) | 0.21(± 0.1) | 167.19(± 29.9) | 436.69(± 60.53) |
| 4 | Acquisition | 400 ms | 450 ms | 450 ms | 49.3 (± 13.0) | 0.3(± 0.11) | 183.24(± 27.97) | 455.63(± 31.51) |
| 5 | Acquisition | 400 ms | 450 ms | 450 ms | 56.6 (±11.1) | 0.36(± 0.1) | 170.41(± 28.7) | 492.65(± 25.28) |
| 6 | Acquisition | 400 ms | 450 ms | 450 ms | 58.5 (10.6) | 0.35(± 0.09) | 203.55(± 26.47) | 470.46(± 26.53) |
| **Main effect session*** | | | | | F(6,4044) = 65.13, p < .00001 | F(6,4044) = 74.82, p < .00001 | F (6,1057) = 1.65, p = 0.13 | F (6,270) = 8.92, p = 6.79E-09 |
|  |  |  |  |  |  |  |  |  |
| 6 | Short CS | 400 ms | 450 ms | 450 ms | 58.46 (± 10.55) | 0.35 (± 0.09) | 203.55 (± 26.47) | 470.46 (± 26.53) |
| 7 | Short CS | 400 ms | 450 ms | 100 ms | 55.77 (± 14.55) | 0.34 (± 0.11) | 172.81 (± 28.47) | 475.53 (± 37.64) |
| **Main effect session*** | | | | | F(1,1169) = 0.57, p=0.45 | F(1,1169) = 0.68, p=0.41 | F(1,387) = 3.64, p=0.06 | F(1,82) = 0.42, p=0.52 |
|  |  |  |  |  |  |  |  |  |
| 6 | ISI switch | 400 ms | 450 ms | 450 ms | 58.46 (± 10.55) | 0.35 (± 0.09) | 203.55 (± 26.47) | 470.46 (± 26.53) |
| 8 | ISI switch | 700 ms | 750 ms | 750 ms | 30.58 (± 15.43) | 0.14 (± 0.15) | 236.78 (± 31.24) | 652.97 (± 91.6) |
| 9 | ISI switch | 700 ms | 750 ms | 750 ms | 40.23 (± 18.71) | 0.22 (± 0.18) | 278.32 (± 78.02) | 765.6 (± 75.85) |
| **Main effect session*** | | | | | F(2,1522) = 65.8, p < .00001 | F(2,1522) = 56.22, p < .00001 | F(2,749) = 3.42, p=0.03 | F(2,113) = 42.21, p < .00001 |
|  |  |  |  |  |  |  |  |  |
| *All values: mean ± 95% confidence interval* | | | | | | | | |
| ** ANOVA on Linear Mixed-Effect model* | | | | | | | | |

| **Supplementary table 3 \| Prepulse inhibition of acoustic startle response (n = 30 participants)** | | | | | | |
| --- | --- | --- | --- | --- | --- | --- |
|  |  |  |  |  |  |  |
| **Trial type** | **Amplitude of eyelid startle in response to pulse** | | | **Amplitude of eyelid startle in response to prepulse** | | |
| Pulse (105 dB) | 0.35 (± 0.08) | | | 0.01 (± 0.02) | | |
| Prepulse 65 dB + Pulse | 0.14 (± 0.06) | | | 0.01 (± 0.03) | | |
| Prepulse 75 dB + Pulse | 0.1 (± 0.05) | | | 0.02 (± 0.02) | | |
| Prepulse 83 dB + Pulse | 0.09 (± 0.04) | | | 0.05 (± 0.03) | | |
| Prepulse 93 dB + Pulse | 0.07 (± 0.03) | | | 0.1 (± 0.03) | | |
| Main effect of trial type* | F(4,1434) = 99.44, p<.00001 | | | F(4,1434) = 15.62, p<.00001 | | |
|  |  | | |  | | |
| **Pairwise differences of trial type** | **Estimate** | **t-ratio** | **p-value**** | **Estimate** | **t-ratio** | **p-value**** |
| (Pulse) vs. (PP 65 dB + Pulse) | 0.203 | 12.758 | <.0001 | -0.0097 | -0.724 | 1 |
| (Pulse) vs. (PP 75 dB + Pulse) | 0.249 | 15.505 | <.0001 | -0.0104 | -0.771 | 1 |
| (Pulse) vs. (PP 83 dB + Pulse) | 0.258 | 15.873 | <.0001 | -0.0477 | -3.483 | 0.0036 |
| (Pulse) vs. (PP 93 dB + Pulse) | 0.279 | 17.291 | <.0001 | -0.0912 | -6.701 | <.0001 |
| (PP 65 dB + Pulse) vs. (PP 75 dB + Pulse) | 0.046 | 2.913 | 0.0145 | -0.0007 | -0.055 | 1 |
| (PP 65 dB + Pulse) vs. (PP 83 dB + Pulse) | 0.054 | 3.423 | 0.0032 | -0.0380 | -2.82 | 0.0244 |
| (PP 65 dB + Pulse) vs. (PP 93 dB + Pulse) | 0.075 | 4.771 | <.0001 | -0.0814 | -6.085 | <.0001 |
| (PP 75 dB + Pulse) vs. (PP 83 dB + Pulse) | 0.008 | 0.535 | 0.5926 | -0.0372 | -2.739 | 0.025 |
| (PP 75 dB + Pulse) vs. (PP 93 dB + Pulse) | 0.029 | 1.851 | 0.1931 | -0.0807 | -5.973 | <.0001 |
| (PP 83 dB + Pulse) vs. (PP 93 dB + Pulse) | 0.021 | 1.298 | 0.3889 | -0.0434 | -3.181 | 0.009 |
|  |  |  |  |  |  |  |
| *All values: mean ± 95% confidence interval* | | | | | | |
| ** ANOVA on Linear Mixed-Effect model* | | | | | | |
| ***P-value adjustment: bonferroni-holm method for 10 tests* | | | | | | |

| **Supplementary Table 4 \| Startle habituation (n = 14 participants)** | | | | | | |
| --- | --- | --- | --- | --- | --- | --- |
|  |  |  |  |  |  |  |
| **Pulse number** | **Percentage eyelid startles** | | | **Eyelid startle amplitude** | | |
| Startle pulse 1 | 39.68 (± 22.47) | | | 0.30 (± 0.15) | | |
| Startle pulse 2 | 27.78 (± 20.4) | | | 0.16 (± 0.14) | | |
| Startle pulse 3 | 21.43 (± 20.05) | | | 0.13 (± 0.15) | | |
| Startle pulse 4 | 21.43 (± 21.12) | | | 0.11 (± 0.14) | | |
| Startle pulse 5 | 19.84 (± 19.19) | | | 0.13 (± 0.14) | | |
| **Main effect of pulse*** | F(4,612) = 9.85, p<.00001 | | | F(4,612) = 18.14, p<.00001 | | |
|  |  | | |  | | |
| **Pairwise differences** | **Estimate** | **t-ratio** | **p-value**** | **Estimate** | **t-ratio** | **p-value**** |
| Startle pulses 1 vs. 2 | 11.9 | 3.215 | 0.0096 | 0.135 | 5.459 | <.0001 |
| Startle pulses 1 vs. 3 | 18.25 | 4.93 | <.0001 | 0.165 | 6.7 | <.0001 |
| Startle pulses 1 vs. 4 | 18.25 | 4.93 | <.0001 | 0.182 | 7.387 | <.0001 |
| Startle pulses 1 vs. 5 | 19.84 | 5.358 | <.0001 | 0.164 | 6.665 | <.0001 |
| Startle pulses 2 vs. 3 | 6.35 | 1.715 | 0.4345 | 0.030 | 1.242 | 1 |
| Startle pulses 2 vs. 4 | 6.35 | 1.715 | 0.4345 | 0.047 | 1.928 | 0.325 |
| Startle pulses 2 vs. 5 | 7.94 | 2.143 | 0.1949 | 0.029 | 1.207 | 1 |
| Startle pulses 3 vs. 4 | 0 | 0 | 1 | 0.017 | 0.687 | 1 |
| Startle pulses 3 vs. 5 | 1.59 | 0.429 | 1 | -0.000 | -0.035 | 1 |
| Startle pulses 4 vs. 5 | 1.59 | 0.429 | 1 | -0.017 | -0.722 | 1 |
|  |  |  |  |  |  |  |
| *All values: mean ± 95% confidence interval* | | | | | | |
| ** ANOVA on Linear Mixed-Effect model* | | | | | | |
| ***P-value adjustment: bonferroni-holm method for 10 tests* | | | | | | |
